# Ligature of the Left Main Coronary Artery after Surgery in Kawasaki Disease: Case Report

**DOI:** 10.21470/1678-9741-2018-0029

**Published:** 2019

**Authors:** Laura Varela Barca, José López-Menéndez, Ana Redondo Palacios, Jorge Rodríguez-Roda Stuart

**Affiliations:** 1 Cardiac Surgery Department, Hospital Ramón y Cajal, Madrid, Spain.

**Keywords:** Mucocutaneous Lymph Node Syndrome, Acute Coronary Syndrome, Coronary Aneurysm, Coronary Artery Bypass, Off-Pump

## Abstract

We report a case of a 23-year-old man who was diagnosed with Kawasaki disease that progressed to a coronary aneurysm in the left main coronary artery (LMA). He had suffered from acute coronary syndrome and then underwent an emergent percutaneous coronary angioplasty, in which a polyurethane-covered stent was placed inside the aneurysm. The stent was thrombosed one year later, despite the patient had been treated with anticoagulant and antiplatelet therapy. Emergency percutaneous intervention was then performed. LMA was reopened and stent malposition was observed. Therefore, urgent coronary bypass grafting was performed in which a high degree of competitive flow was observed through the reopened stent. LMA was ligated at the inflow of the aneurysm, resulting in an improvement of graft flow. Left main ligature has not been previously reported.

**Table:** 

Abbreviations, acronyms & symbols	
ACS	= Acute coronary syndrome
CABG	= Coronary artery bypass grafting
IMA	= Internal mammary arteries
LAD	= Left anterior descending coronary artery
LCX	= Left circumflex artery
LMA	= Left main coronary artery
PI	= Pulsatility index
RCA	= Right coronary artery

## INTRODUCTION

Kawasaki disease is a type of vasculitis that affects small and medium-sized arteries with predilection for coronary arteries. It is more prevalent in Asian countries, and the most significant complication is the development of coronary artery aneurysms that occur between 15-25% of the cases.

Approximately, 50% of coronary artery aneurysms regress, but other moderately sized aneurysms can lead to clinical problems even years after an acute episode of Kawasaki disease^[1]^. Although the death rate associated with Kawasaki disease is very low (<0.1%), myocardial infarction due to stenotic lesions or thrombosis of aneurysms continues to be a serious problem, despite diagnostic and therapeutic advances^[2]^.

Percutaneous coronary angioplasty is indicated in localized stenotic lesions not involving the coronary ostia, but coronary artery bypass grafting (CABG) should be recommended in cases with myocardial ischemia caused by multivessel disease^[3]^.

We report a case of urgent CABG in a patient diagnosed with Kawasaki disease, despite adequate flow through a stent in the left main coronary artery (LMA). Competitive flow through coronary grafts significantly decreases the grafts' durability and could facilitate graft thrombosis^[4]^. There are previously published reports about the ligature of the right coronary artery (RCA) after CABG^[5]^, but to our knowledge the ligature of LMA has not been previously reported.

## CASE REPORT

This case report was prepared following the CARE Guidelines^[6]^ and the patient provided informed consent for its publication.

We present a case of a 23-year-old man who was diagnosed with Kawasaki disease at the age of 13 months when he suffered an acute coronary syndrome (ACS). Coronary artery aneurysms in RCA and LMA were observed, as well as partial thrombosis in both. Accordingly, he was treated with fibrinolysis and anticoagulant therapy. Complete regression of the RCA aneurism was observed six years after the diagnosis of Kawasaki disease, however the LMA aneurysm persisted with involvement of the anterior descending artery ostium.

**Fig. 1:**
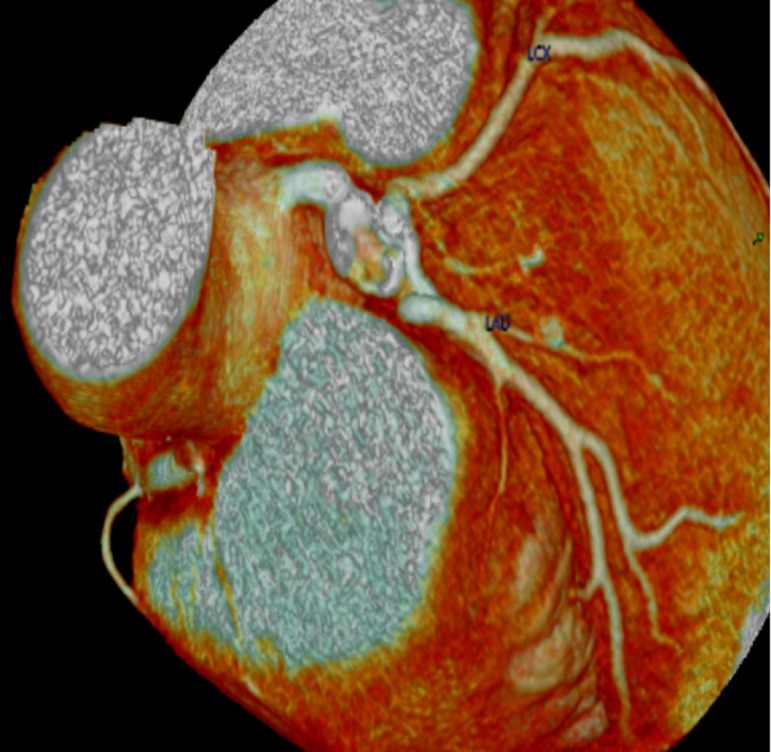
Coronary artery computed tomographic reconstruction showing an aneurysmatic lesion of the left main artery. LAD=left anterior descending coronary artery; LCX=left circumflex artery

One year before the current episode, the patient suffered from ACS due to a severe calcified lesion of the LMA aneurysm (Figure 1). He underwent an emergency percutaneous coronary angioplasty, and a polyurethane-covered stent (PK Papyrus, Biotronik(r), Berlin, Germany) was inserted. The patient remained asymptomatic for one year.

**Fig. 2:**
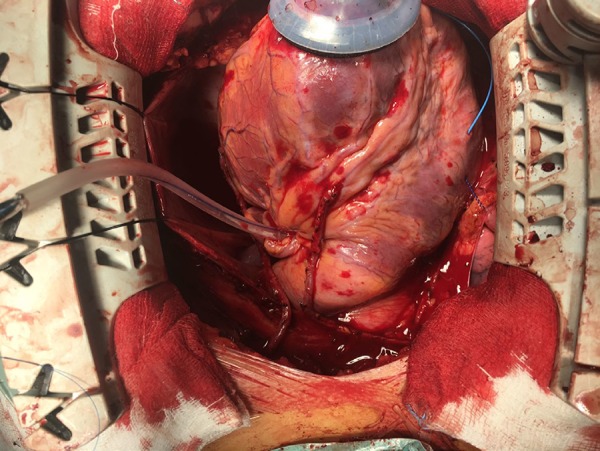
Image of the surgical field: testing with a transitory tourniquet on the left main artery, prior to permanent ligature.

In the current episode, he was readmitted to the emergency department in cardiogenic shock in which noradrenalin employment was necessary to achieve hemodynamic stabilization. He suffered from ACS caused by a complete occlusion of the stent despite anticoagulation and dual antiplatelet therapy. It was possible to percutaneously reopen the occluded LMA and adequately restore coronary flow. However, there was evident severe stent malposition in the distal part of the LMA. Due to this concerning finding, the patient was accepted for emergency cardiac surgery. He underwent off-pump CABG 24 hours after the episode, in which both internal mammary arteries (IMA) were dissected. The right IMA was grafted to the left medial anterior descending artery, and the left IMA was grafted to the first marginal artery. A transit-time flow meter was employed to assess graft patency (Medistim(r), Oslo, Norway). Although flow measures and pulsatility index (PI) were optimal (60 ml/min in the right IMA graft and 50 ml/min in the left IMA graph, with a PI of 2.4 and 75% insufficiency), a high degree of competitive flow was observed through the recently reopened LMA. A transitory tourniquet was applied to the LMA (Figure 2) and there was great improvement in the flow through the grafts with complete disappearance of any competitive flow (150 ml/min and 80 ml/min in right and left IMA, respectively, and insufficiency decreased to 0%). Therefore, the LMA was ligated at the inflow of the aneurysm.

The patient was discharged after 24 hours in intensive care after surgery and a total stay of six days. The patient has remained asymptomatic since the procedure with a post-operative follow-up at 16 months to date.

## DISCUSSION

Surgical revascularization for coronary lesions secondary to Kawasaki disease is relatively uncommon. However, CABG should be recommended in cases of patients with multivessel disease.

According to the current guidelines^[3]^, complete arterial revascularization should be used in young patients with small comorbidities in order to achieve good long-term graft patency. Left IMA should be used to bypass the left anterior descending artery, and one second IMA should be indicated to graft the left circumflex or right coronary in order to improve the likelihood of survival and to decrease reintervention^[3]^.

Off-pump CABG has been previously described as treatment for coronary artery aneurysms^[5,7]^. Off-pump surgery is a safe and less invasive procedure because it minimizes the complications of cardiopulmonary bypass^[8]^.

Since three decades ago, IMA graft was first described rather than saphenous vein graft during CABG^[9]^; IMA are the preferred conduits for surgical revascularization. They have confirmed excellent long-term patency due to their endothelial function^[10]^. IMA grafts have shown improved survival and reduced incidence of myocardial infarct, recurrent angina, and the need for repeated intervention^[3,11]^.

In addition, considering that early thrombus formation in saphenous vein grafts has previously been reported, surgery using arterial grafts should be considered. Although in cases of non-severe stenosis in the native coronary arteries, the use of IMA is widely debated^[4]^. It is known that competitive blood flow reduces IMA graft patency. This reduction was described to be particularly evident in diastole^[12]^.

To avoid the risk of graft failure due to highly competitive flow we performed a transient ligature in LMA. There was great improvement of flow through the grafts, with complete disappearance of competitive flow. Therefore, the LMA was ligated at the inflow of the aneurysm. Since there is an absence of reports in literature about LMA ligation, we believe that this technique is a good option to avoid competitive flow through a patent calcified giant aneurysm after Kawasaki disease.

**Table:** 

Authors' roles & responsibilities	
LVB	Substantial contributions to the conception or design of the work; or the acquisition, analysis, or interpretation of data for the work; drafting the work or revising it critically for important intellectual content; final approval of the version to be published
JLM	Acquisition of information and design; final approval of the version to be published
ARP	Design and images; final approval of the version to be published
JRRS	Review of the document; final approval of the version to be published
